# Molecular Mapping of Hydrogen Sulfide Targets in Normal Human Keratinocytes

**DOI:** 10.3390/ijms21134648

**Published:** 2020-06-30

**Authors:** Olivia Gross-Amat, Marine Guillen, Jean-Pascal Gimeno, Michel Salzet, Nicolas Lebonvallet, Laurent Misery, Céline Auxenfans, Serge Nataf

**Affiliations:** 1 Lyon-Est School of Medicine, University Claude Bernard Lyon-1, 69100 Villeurbanne, France; marine.guillen@univ-lyon1.fr (M.G.); serge.nataf@inserm.fr (S.N.); 2 Bank of Tissues and Cells, Lyon University Hospital (Hospices Civils de Lyon), 69003 Lyon, France; celine.auxenfans@chu-lyon.fr; 3 CarMeN Laboratory, INSERM U1060, INRA U1397, INSA de Lyon, 69600 Oullins, France; 4 Inserm, CHRU Lille, U-1192-Laboratoire Protéomique, Réponse Inflammatoire et Spectrométrie de Masse-PRISM, University of Lille, F-59000 Lille, France; jean-pascal.gimeno@univ-lille.fr (J.-P.G.); michel.salzet@univ-lille.fr (M.S.); 5 Laboratory of Epithelial-Neural Interactions, University of Brest, LIEN, 29200 Brest, France; nicolas.lebonvallet@univ-brest.fr (N.L.); laurent.misery@chu-brest.fr (L.M.); 6 Department of dermatology, Brest University Hospital (CHU de Brest), 29200 Brest, France; 7 Tissue Biology and Therapeutic Engineering Laboratory, UMR 5305, 69007 Lyon, France

**Keywords:** human keratinocyte, H_2_S, RNAseq, proteomics

## Abstract

Although sulfur-rich thermal waters have ancestrally been used in the context of dermatological conditions, a global mapping of the molecular effects exerted by H_2_S on human keratinocytes is still lacking. To fill this knowledge gap, we subjected cultured human keratinocytes to distinct amounts of the non-gaseous hydrogen sulfur donor NaHS. We first checked that H_2_S accumulated in the cytoplasm of keratinocytes under our experimental conditions andused a combination of proteomics, genomics and biochemical approaches to unravel functionally relevant H_2_S targets in human keratinocytes. We found that the identified targets fall into two main categories: (i) the oxidative stress response molecules superoxide dismutase 2 (SOD2), NAD(P)H quinone dehydrogenase 1 (NQO1) and culin 3 (CUL3) and (ii) the chemokines interleukin-8 (IL-8) and CXCL2. Interestingly, NaHS also stimulated the caspase-1 inflammasome pathway, leading to increased secretion of the pro-inflammatory molecule interleukin-18 (IL-18). Interestingly, the secretion of interleukin-1 beta (IL-1β) was only modestly impacted by NaHS exposure despite a significant accumulation of IL-1β pro-form. Finally, we observed that NaHS significantly hampered the growth of human keratinocyte progenitors and stem cells cultured under clonogenic conditions or as epidermal cell sheets. We conclude that H_2_S exerts specific molecular effects on normal human keratinocytes.

## 1. Introduction

Sulfur-rich thermal waters are an ancestral dermatological therapy which nowadays is used as an adjunct treatment for psoriasis and atopic dermatitis [1,2,3,4] but also chronic skin wounds [4,5]. Hydrogen sulfide (H_2_S), the main bioactive component specific to sulfur-rich thermal waters, belongs to the small family of physiological gaseous transmitters, which also comprises nitric oxide (NO) and carbon monoxide (CO). Similarly to NO and CO, H_2_S is endogenously produced and exerts potent signaling effects, but exhibits a short half-life and a rapid decrease in its concentration at the site of production or application. Such a volatility renders the use of H_2_S difficult under *in vitro*or *in vivo* experimental conditions. As a consequence, the non-gaseous hydrogen sulfur donor NaHS is frequently preferred for research purposes. In order to mimic the impact of sulfur-rich spa waters on skin cells, previous works thus assessed the effects of NaHS on the proliferation, differentiation, adhesion properties and cytokine profile of cultured human keratinocytes [6,7,8,9,10]. Under these experimental conditions, NaHS was notably reported to inhibit the synthesis of anti-inflammatory molecules such as IL-8 and IL-1β, which provided support for the use of sulfur-rich thermal waters for the treatment of psoriasis [7,9]. However, as such findings were obtained from transformed keratinocyte cell lines, their physiological relevance remains to be confirmed in primary cultures of keratinocytes. Moreover, none of the above-mentioned works used systems biology approaches to assess, other than a priori, the global impact of NaHS on human keratinocytes. Hence, a systematic molecular mapping of the effects exerted by NaHS on human primary cultures of keratinocytes is still missing. To fill this knowledge gap, we performed here a pan-genomics and pan-proteomics analysis of cultured human keratinocytes exposed to NaHS. Our results show that NaHS inhibits the proliferation of human keratinocyte progenitors and stem cells, stimulates their secretion of specific pro-inflammatory cytokines and promotes the synthesis of molecules involved in antioxidative response. These findings provide insights into the molecular effects exerted by sulfur-rich spa waters on skin cells and point to the potential role of H_2_S as a gaseous regulator of epidermal cell homeostasis.

## 2. Results

### 2.1. NaHS Induces a Rapid and Transient Increase in H_2_S in Human Keratinocyte Cultures

**Figure 1:**
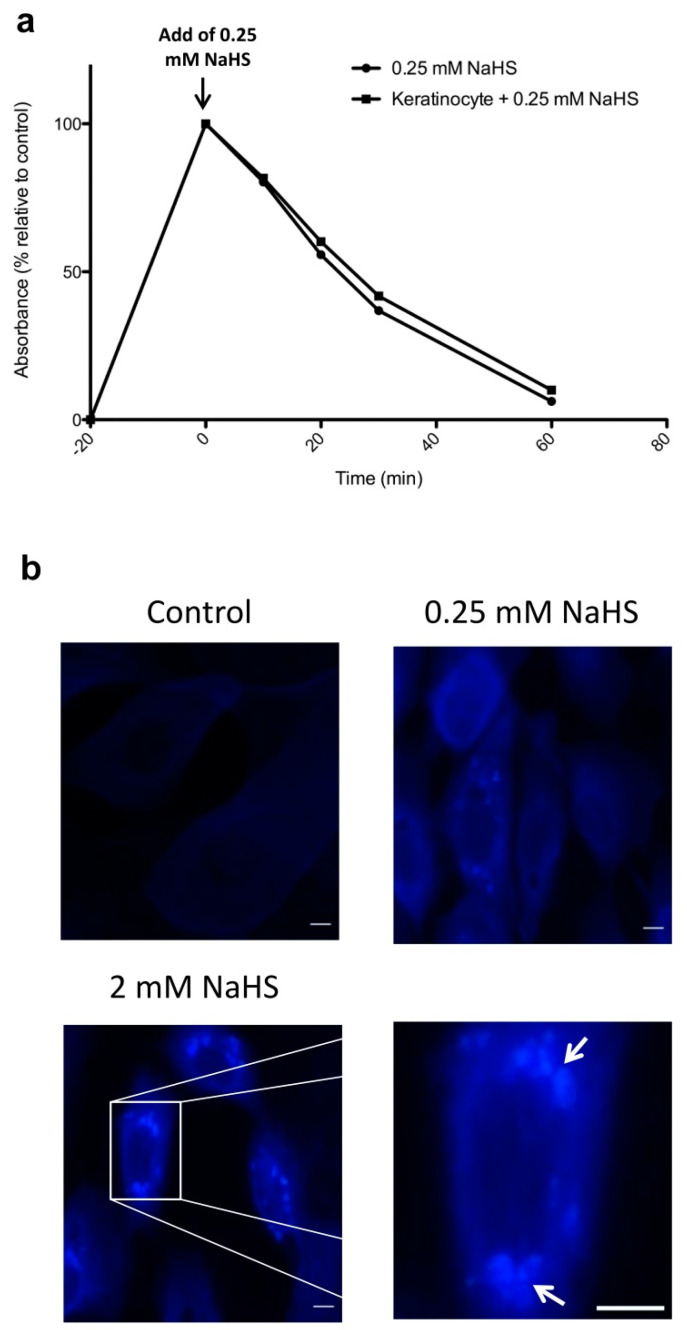
NaHS induces the extracellular and intracellular accumulation of H_2_S in human keratinocyte cultures. (**a**) Kinetics of H_2_S accumulation in keratinocyte culture medium following addition of 0.25 mM NaHS in the presence or absence of cultured keratinocytes. (**b**) The use of a fluorescent H_2_S probe allowed detection of H_2_S accumulation (arrows) in the cytoplasm of cultured keratinocytes stimulated with NaHS at a concentration of 0.25 or 2 mM. Results are representative of three to five experiments. Scale bar: 0.01 mm.

To assess the actual impact of NaHS on the generation of H_2_S in our experimental conditions, we first measured H_2_S amounts in the supernatant of control vs. NaHS-treated cultures of human keratinocytes. We found that NaHS added to the culture media at a concentration of 0.25 mM induced a quick rise in H_2_S, irrespective of the presence or absence of cultured keratinocytes (Figure 1a). As a return to baseline levels was observed 1 h after NaHS exposure (Figure 1a), these results demonstrate that NaHS exerts rapid and transient effects on H_2_S amounts measured in the culture media. In parallel experiments, we also sought to determine whether, as a consequence of an H_2_S rise in the culture medium, an intracellular increase in H_2_S could be observed in the cytoplasm of cultured human keratinocytes. The use of a fluorescent H_2_S probe allowed us to demonstrate that NaHS-treated keratinocytes exhibited an intracytoplasmic accumulation of H_2_S, which could be observed 1h following NaHS exposure (Figure 1b and Data Supplement Figure S1). Interestingly, such an effect appeared to be more pronounced when higher concentrations of NaHS were applied (Figure 1b).

### 2.2. NaHS Impairs the Growth of Human Keratinocyte Progenitors and Stem Cells

**Figure 2:**
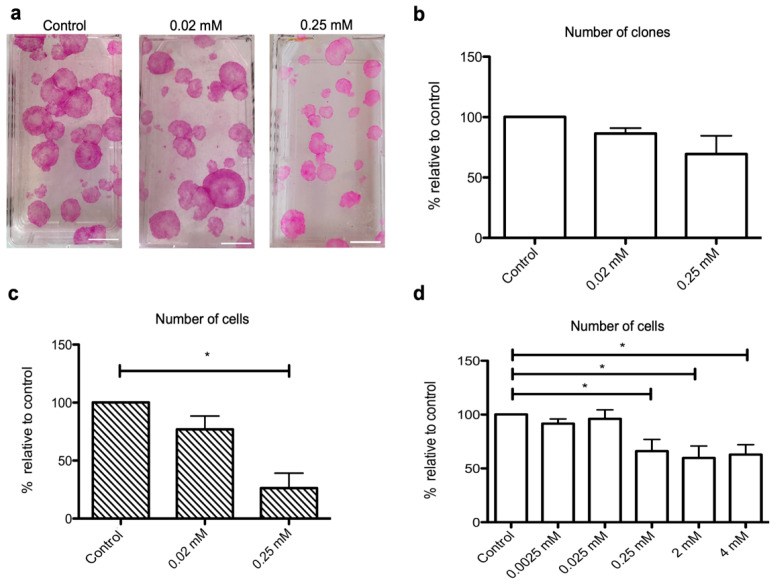
NaHS impairs the growth of human epidermal stem cells and epidermal cell sheets. (**a–c**) Human keratinocytes cultured under clonogenic conditions were stimulated every other day with NaHS at 0.02 or 0.25 mM concentrations during 12 days. Cells were then fixed and stained with hematein–eosin, allowing colonies of keratinocyte stem or progenitor cells to be manually counted. Keratinocyte cultures generated in parallel under strictly identical conditions were trypsinized, and living cells were numerated following trypan blue staining. Four keratinocyte cultures deriving from four distinct donors were used in these experiments. (**a**) Representative images of grown colonies obtained under control condition (upper panel) or under NaHS stimulation at 0.02 mM concentration (middle panel) or 0.25 mM concentration (lower panel). Images show that, as compared to control untreated cells or cells treated with 0.02 mM NaHS, colonies are of lower size in cells treated with 0.25 mM NaHS (**b**) Irrespective of NaHS concentration, the total number of colonies remains unchanged in NaHS-treated cells as compared to control cells. (**c**) However, the total number of harvested living cells is significantly lower in cultures treated with 0.25 mM NaHS as compared to control untreated cultures. (**d**) The effects of NaHS on the cellular density of epidermal cell sheets were assessed after 24h treatment with NaHS at concentrations ranging from 0.0025 mM to 4 mM. Experiments were performed on cells obtained from six distinct donors. Results are expressed as percentages relative to control conditions. Statistical significance of paired comparisons was assessed with the Wilcoxon test. *: *p* < 0.05. Scale bar: 1 cm.

To assess the impact of NaHS on the growth of keratinocyte progenitors and stem cells, we performed a keratinocyte clonogenic assay (CFU) and measured the effects of NaHS (0.02 mM or 0.25 mM) on both the number and size of colonies. While the number of clones remained unchanged under NaHS treatment (Figure 2a,b), we observed that the clones exhibited smaller sizes (Figure 2a). Accordingly, the counts of harvested cells obtained 10–14 days post-treatment were nearly 80% lower in cultures treated with 0.25 mM NaHS as compared to controls (Figure 2c). Such an effect was not observed when 0.02 mM NaHS was applied. Similarly, in cultures of human epithelial cell sheets, which are composed of more than 90% CD49f-positive keratinocyte progenitors [11,12] (Data Supplement, Figure S2 and Table S1), total cell counts were significantly lower in NaHS-treated cultures as compared to controls (Figure 2d). However, such a growth-limiting effect was less pronounced under these experimental conditions and reached only 30% in NaHS-treated as compared to control cultures. Again, NaHS applied at concentrations below 0.25 mM had no impact on the growth of keratinocyte progenitors. Of note, irrespective of the tested NaHS concentrations (from 0.0025 to 4 mM), we did not observe any impact on keratinocyte cell viability as assessed by trypan blue coloration or MTT (3-(4,5-dimethylthiazol-2-yl)-2,5-diphenyltetrazolium bromide) assay (Data Supplement, Figure S3).

### 2.3. NaHS Stimulates the Synthesis of Superoxide Dismutase 2 (SOD2) by Cultured Human Keratinocytes

We used liquid chromatography–mass spectrometry (LC-MS) to perform a pan-proteomic analysis of the effects exerted by 0.25 mM NaHS on human epidermal cell sheets. It should be noticed that LC-MS is known to be a relatively poorly sensitive technique allowing the detection of only abundantly expressed proteins. Accordingly, only 337 proteins constantly reached the threshold of detection in the analyzed samples, and paired comparisons showed that 10 proteins exhibited differential expression with p-values <0.05 (Data Supplement, Table S2). However, fold changes were relatively low (<1.5), and statistical significance was not confirmed when p-values were adjusted with the Benjamini–Hochberg procedure. We nevertheless combined these results with a manual curation of the literature to identify candidate molecules which would be worth assessing with more sensitive techniques. Among the 10 identified candidate molecules, only superoxide dismutase 2 (SOD2) was previously shown to be regulated by NaHS. SOD2 is an antioxidant enzyme which decreases the level of ROS (reactive oxygen species), thus participating in cellular defense against oxidative stress. On this basis, we thus performed an ELISA measurement of SOD2 on cell extracts derived from NaHS-treated or control epidermal cell sheets and observed that NaHS induced a nearly 100% increase in SOD2 amounts in NaHS-treated epidermal cell sheets (*p* < 0.05) (Data Supplement, Figure S4). To further assess the links between NaHS and oxidative stress, we then performed a complementary bioinformatics analysis based on a survey of the comparative Toxocogenomics database (CTD) [13]. This approach allowed us to retrieve a list of 504 genes (Data Supplement, Table S3), which, in human cells, were previously reported to be modulated by hydrogen-sulfide-containing compounds. Confirming our proteomics data on SOD2, the highest functional enrichment retrieved from this list was obtained with genes annotated with the GO term “response to reactive oxygen species” (Table 1).

### 2.4. NaHS Exerts Specific Effects on the RNA Profile of Cultured Human Keratinocytes

We then performed an RNA-seq analysis to assess the genomic impact of 0.25 mM NaHS on human epidermal cell sheets. While 12.000 to 14.000 transcripts were detected in each sample, paired comparisons allowed identifying only 265 protein-coding differentially expressed genes with a fold change >1.5 and an adjusted *p*-value < 0.05 (Data Supplement, Table S4). Among these 265 genes, 122 were up-regulated and 143 were down-regulated by NaHS treatment. While such lists were not enriched in genes signing specific molecular pathways according to the enrichment analysis platform Enrichr, 11 of the identified genes (Table 2) belonged to the list of 504 hydrogen sulfide target genes we retrieved from the CTD database. These notably comprised the oxidative stress response genes NQO1 (NAD(P)H Quinone Dehydrogenase 1) [15] and CUL3 (Culin 3) [16], the chemokine CXCL2 (C-X-C Motif Chemokine Ligand 2) [17,18,19,20] and the inflammasome component CASP1 (Caspase-1), which enables the maturation of IL-1β (Interleukin-1β) and IL-18 (Interleukin-18) [21,22].

### 2.5. NaHS Modifies the Secretary Profile of Cultured Human Keratinocytes

**Figure 3:**
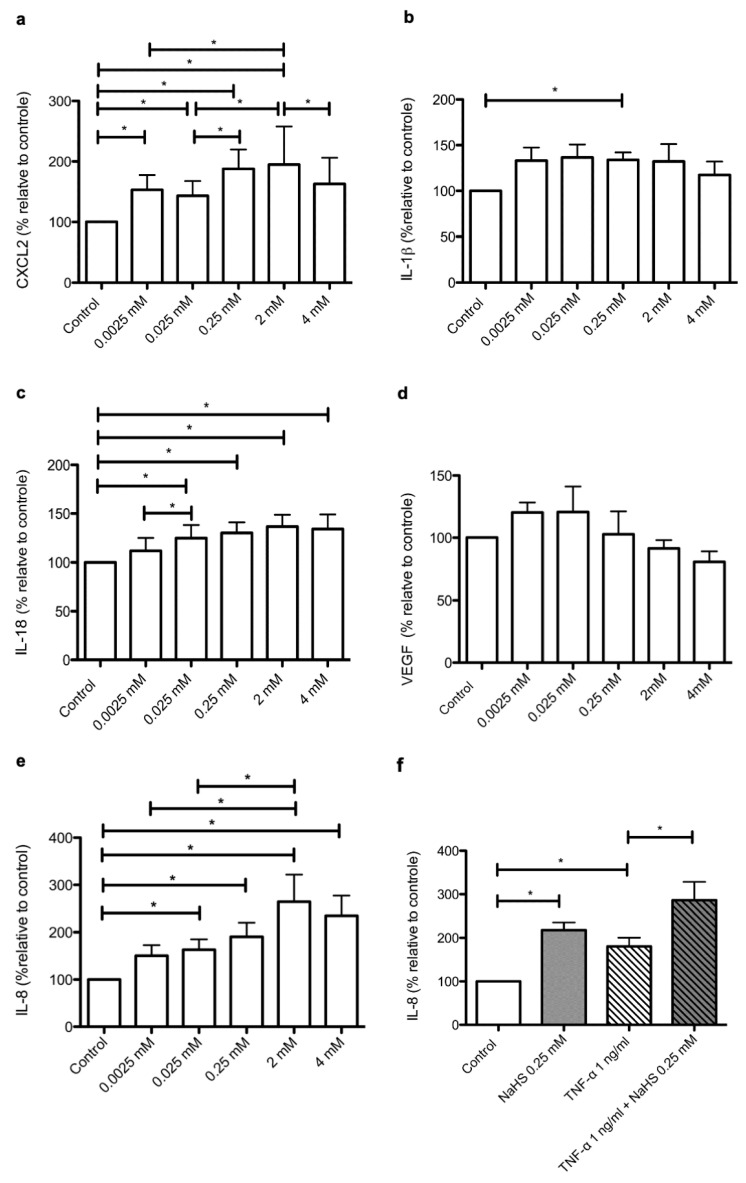
NaHS treatment modifies the cytokine/chemokine secretion profile of human epidermal cell sheets. Human epidermal cell sheets were cultured under control conditions or stimulated with NaHS at 0.0025, 0.025, 0.25, 2, or 4mM, alone or in combination with TNF-α (1 ng/mL Cell supernatants were then recovered 24 h after stimulation and the cytokines CXCL2 (**a**), IL-1β (**b**), IL-18 (**c**), VEGF (**d**) and IL-8 (**e**–**f**) were measured by ELISA. Results are expressed as percentages relative to control measures observed in the absence of NaHS and/or TNF-α stimulation. Experiments were performed on epidermal cell sheets obtained from at least five distinct donors. The statistical significance of paired comparisons was assessed with the Wilcoxon test. *: *p* < 0.05.

The results we obtained by RNA-seq analysis prompted us to investigate, at the protein level, the impact of NaHS on the secretion of specific components of the cytokine/chemokine inflammatory response. Besides CXCL2, IL-1 beta (IL-1β) and IL-18, we focused our analysis on IL-8 (interleukin-8) and VEGF (vascular endothelial growth factor), two cytokines highly expressed by human keratinocytes under basal conditions [23,24,25,26]. We observed that NaHS, when applied at the concentration of 0.25 mM, induced a statistically significant increase in IL-1β, IL-18, IL-8 and CXCL2 (Figure 3). However, a significant dose-dependent effect of NaHS was demonstrated only for the synthesis of IL-8 (Figure 3e) and CXCL2 (Figure 3a). Moreover, as compared to IL-18 (Figure 3c) and IL-1β (Figure 3b), the stimulating impact of NaHS was far more pronounced on IL-8 (maximal increase: 160%) and CXCL2 (maximal increase: 90%). Indeed, NaHS at a concentration of 0.25 mM stimulated IL-8 secretion at similar levels to the prototypical pro-inflammatory cytokine TNF-⍺ (tumor necrosis factor alpha) [27,28] (Figure 3f). In addition, NaHS and TNF-⍺ exerted synergistic stimulating effects on the synthesis of IL-8 (Figure 3f). Such findings remained unchanged when taking into account the impact of NaHS on the growth of epidermal cell sheets (Data Supplement, Figure S5). In contrast with the impact of NaHS on IL-8 secretion, NaHS induced an only limited increase in IL-1β synthesis. Of note, such an effect was not dose-dependent and was exclusively observed for a concentration of 0.25 mM NaHS. Moreover, maximum mean values measured for IL-1β remained below 8 pg/mL, compared to more than 6000 pg/mL for IL-8, nearly 1000 pg/mL for IL-18 and more than 60 pg/mL for CXCL2 (Data Supplement, Figure S6). Finally, NaHS had no measurable effect on the synthesis of VEGF (Figure 3d), a molecule sharing functional properties with IL-8 with regard to its pro-angiogenic effects. Altogether, these data demonstrate that NaHS modifies, in a specific manner, the chemokine/cytokine secretome of cultured human keratinocytes.

### 2.6. Distinct Molecular Pathways Mediate the Stimulating Effects of NaHS on the Synthesis of IL-1β and IL-18 by Cultured Human Keratinocytes

**Figure 4:**
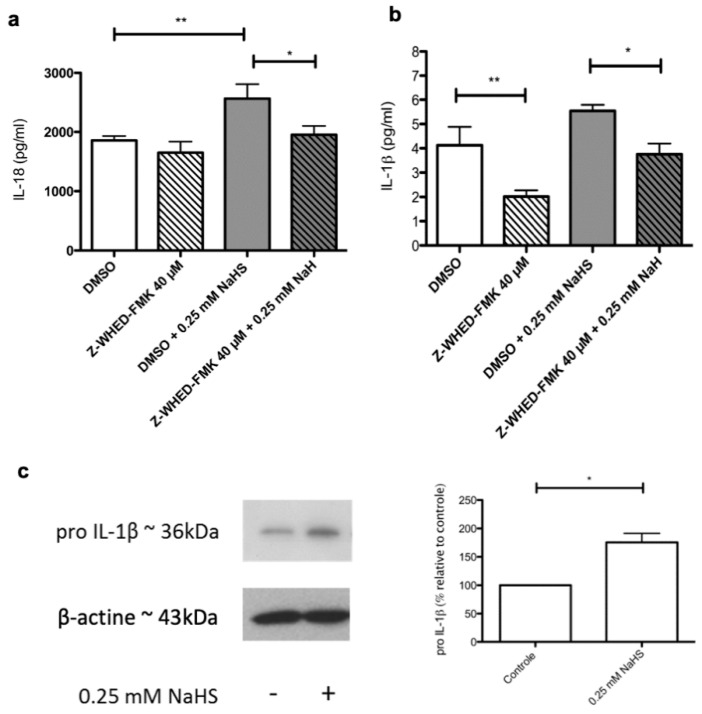
Role of the caspase-1 inflammasome pathway in NaHS-induced synthesis of IL-18 and IL-1β. Human epidermal cell sheets were stimulated with NaHS at 0.25 mM in the presence of either the caspase-1 inhibitor Z-WHED-FMK 40 µM diluted in DMSO or DMSO alone (**a**,**b**). Cell supernatants were then recovered 24h after stimulation and the cytokines IL-18 (**a**) and IL-1β (**b**) were measured by ELISA. In other experiments, the amount of IL-1β pro-form was measured by western blotting on protein extracts obtained from control or NaHS-treated human epidermal cell sheets (**c**). Experiments were performed three to five times on human epidermal cell sheets derived from one donor. Statistical significance of unpaired comparisons was assessed with the Mann and Whitney test.*: *p* < 0.05; **: *p* < 0.01.

To start getting insights into the molecular pathways involved in NaHS-induced alterations of the secretome of cultured human keratinocytes, we assessed whether a pharmacological inhibition of human caspase-1 could block, at least partially, the stimulating effects exerted by NaHS on IL-1β and IL-18. We observed that, as expected, the stimulating effect of NaHS on IL-18 was significantly dampened in the presence of the caspase-1 inhibitor Z-WHED-FMK (Figure 4a). In contrast, this was not observed regarding IL-1β synthesis (Figure 4b). It should be noticed that such a negative result might be explained by two combined parameters: i) the relatively low amplitude of the stimulating impact of NaHS on IL-1β secretion and ii) the fact that dimethysulfoxide (DMSO), used as a vehicle for the caspase-1 inhibitor Z-WHED-FMK, has itself exhibited a slight stimulating effect on IL-1β secretion. Finally, a western blot analysis showed that NaHS increased the levels of intracytoplasmic IL-1β proform (Figure 4c), indicating that mechanisms complementing the caspase-1/inflammasome pathway may support the pro-inflammatory effects exerted by NaHS on cultured human keratinocytes.

## 3. Discussion

Most of the results presented in this paper were obtained with a concentration of 0.25 mM NaHS, i.e., 14 mg/L NaHS. Since each NaHS molecule generates one H_2_S molecule, the expected concentration of H_2_S corresponding to 0.25 mM NaHS is likely to stay in the concentration ranges previously measured in sulfur-rich spa waters, i.e., 0.5 to 20 mg/L [29]. We also demonstrated that a single stimulation with NaHS was sufficient to induce the subsequent accumulation of H_2_S in the cytoplasm of cultured keratinocytes. These observations thus support the relevance of our experimental setting to study the molecular impact of sulfur-rich spa waters on human skin cells.

It should be noted that, among cells of the keratinocyte lineage, only keratinocyte stem cells and progenitors are endowed with the ability to proliferate under physiological conditions. Nevertheless, to our knowledge, the few works reporting on the effects of NaHS on the proliferation of human keratinocytes were performed exclusively on transformed or cancerous keratinocyte cell lines [6,8,10]. Using two distinct experimental settings, i.e., clonogenic cultures of keratinocytes and cultures of epidermal cell sheets, we found that NaHS hampered the growth of keratinocyte progenitors and stem cells. These findings contradict previous results obtained with keratinocyte cell lines [6,8] and provide a possible explanation for the reported beneficial effects exerted by sulfur-rich spa waters in patients suffering from psoriasis, a keratinocyte hyperproliferative skin disease. 

The present paper is also the first work reporting on a global mapping, at both the mRNA and protein levels, of the molecular effects exerted by NaHS on cultured human keratinocytes. Our data show that human keratinocytes exposed to NaHS engage an oxidative stress response involving SOD2, NQO1 and CUL3. We also report here that the whole list of currently known targets of sulfide-rich compounds is highly significantly enriched in antioxidant stress response genes. Accordingly, in other models and cell types, NaHS was similarly found to induce an oxidative stress response via the up-regulation of SOD1, SOD2, CAT (Catalase) and/or GPX (glutathione persoxydase 1) [30,31,32]. More specifically, previous reports demonstrated in non-skin cells that the NaHS-induced up-regulation of *NQO1* and *SOD2* mRNAs [33,34] was mediated by the transcription factor NFEL2 (nuclear factor erythroid 2 like 2, also known as NRF2), a key orchestrator of the genomic oxidative stress response [35,36,37]. In this view, based on the well-established role of cullin-3 as a promoter of NRF2 degradation [34,38,39], the NaHS-induced up-regulation of *CUL3* mRNA levels in human keratinocytes might reflect a negative feedback loop aimed at terminating the oxidative stress response. In any case, the primary molecular mechanisms linking H_2_S to oxidative stress remain poorly understood. Of note, any intracytoplasmic elevation of H_2_S levels results in the generation of reactive sulfur species (RSS), which, just as reactive oxygen species (ROS), trigger an anti-oxidative stress response [40]. In a rather provocative view, a recent paper proposed that in archaic forms of life, coping with a sulfur-rich environment was the primary function of genes which are now devoted to protect cells from oxidative stress [41].

Besides oxidative-stress-related molecules, we also found that, in epidermal cell sheets, NaHS specifically promoted the secretion of IL-8 and CXCL2, two cytokines/chemokines constitutively expressed by keratinocytes [25,26,42] and involved in both skin inflammatory responses [17,19] and skin repair [43,44]. In contrast, NaHS did not promote or only modestly promoted the synthesis of molecules that are frequently up-regulated under skin inflammatory conditions, namely VEGF and IL-1β. Overall, the impact of NaHS on the secretory profile of human keratinocytes is specific and finely tuned and appears to involve multiple mechanisms operating at the transcriptional, translational and, possibly, post-translational levels.

Our work provides experimental evidence that H_2_S contained in sulfur-rich spa waters is a bio-active component able to modify the immunological status of non-diseased human keratinocytes. Further studies are needed to establish causal links between the above-identified biological effects and the previously reported medical benefits afforded by sulfur-rich spa waters in conditions such as psoriasis, eczema or wound healing [1,2,3,4]. In particular, it would be interesting to assess the effects of NaHS on keratinocytes derived from psoriatic lesions.

## 4. Materials and Methods

### 4.1. Ethical Statement

Samples were anonymized, and written informed consent was obtained in accordance with the ethical guidelines of Lyon University Hospital (Hospices Civils de Lyon) and the principles of the Declaration of Helsinki. All the samples used in this study belong to a collection of human skin samples declared to the French research ministry (Declaration no. DC-2008-162), delivered to the Bank of Tissues and Cells of the Hospices Civils de Lyon.

### 4.2. Generation of Cultured Human Epidermal Sheets

Normal human epidermal keratinocyte were grown on a feeder layer of irradiated human fibroblasts as previously described [45,46] in keratinocyte culture medium (KCM). See the Supplementary Data for further details. When needed, cells were harvested with trypsin-EDTA 0.05% (Thermo Fisher Scientific, Waltham, MA, USA) and collected for analysis.

### 4.3. NaHS

For each experiment, a fresh solution of 0.25 M NaHS (Sigma-Aldrich, St. Louis, MO, USA) dissolved in PBS was prepared and used as a stock solution to perform ad-hoc primary dilution in DMEM supplemented with 0.1% FCS. Depending on the desired final concentrations, secondary dilutions were then performed in cell culture medium.

### 4.4. Measurements of H_2_S in Culture Supernatants

H_2_S concentrations were measured in culture medium as previously described [47,48] with slight modifications. Briefly, supernatants were collected at different time points (0–10–20–30–60 min–4 h–24 h) following the addition of 0.25 mM NaHS to the cell culture medium. A quantity of 500 µL of harvested supernatant was then mixed with 250 µL of zinc acetate (1% *w*/*v*), and 133 µL of *N*,*N*-dimedthyl-*p*-phenulenediamine sulfate (20 mM) in 7.2 M HCl was then added followed by 133 µL of FeCl_3_ (30 mM) in 1.2 M HCl and 250 µL of trichloroacetic acid (10%, *w*/*v*). Within 15 min thereafter, absorbance was measured at 670 nm. In parallel, a calibration curve was obtained from the analysis of defined H_2_S concentrations (5–20–50–100–250 µM).

### 4.5. Fluorescence-Based Detection of Intracellular H_2_S

Keratinocytes were cultured until sub-confluence on cover glasses in a 24-well plate. NaHS (0.25 or 2 mM) and H_2_S fluorescent probes (100 µM, P3, Sigma) were co-incubated for 1 h at 37 °C and 5% CO_2_. Cells were rinsed 3 times in PBS 1X and fixed in 4% formaldehyde. Cover glasses were then transferred on a slide. Detection of intracellular fluorescence was carried out on an Eclipse 50i microscope (Nikon, Champigny sur Marne, Val-de-Marne, France).

### 4.6. Clonogenic Assay

A keratinocyte clonogenic assay was performed as previously described following a procedure routinely used in our laboratory [49]. See the Supplementary Data for further information. Experiments were performed on cells derived from five independent donors and a total of 12 flasks per donor (6 treated and 6 untreated) were analyzed.

### 4.7. RNA–seq and Proteomics Analyses

Epidermal cell sheets were generated by culturing human keratinocytes for 12 days as described above. Epidermal cell sheets were then treated or not for 24 h with 0.25 mM NaHS then lysed using a RLT lysis buffer (Qiagen, Hilden, Germany) for RNA-seq analyses or mechanically detached using a cell scraper for proteomics analyses. RNA-seq and proteomics analyses were performed by the Lyon-1 university genomics platform ProfileXpert and the Lille university proteomics platform PRISM (Proteomics Inflammatory Response Mass Spectrometry, INSERM U1192), respectively, as previously described [50,51,52,53] and detailed in the Supplementary Data.

### 4.8. Enzyme-Linked Immunosorbent Assay (ELISA)

Standard procedures were applied and are detailed in the Supplementary Data.

### 4.9. Western Blot Analysis

Standard procedures were applied and are detailed in the Supplementary Data. To ensure that equal loading had been performed, membrane stainings with amido black were assessed as previously described [54]. Alternatively, membranes were incubated with an antibody against β-actin (1:2000, sc-47778, Clone C4, Santa Cruz Biotechnology, Inc, Heidelberg, Germany).

### 4.10. Statistical Analysis

Quantitative data obtained by ELISA or western blotting were generated from at least 5 independent experiments in which NaHS-stimulated cells were compared to control untreated cells. Statistical significance was calculated using the paired Wilcoxon test. In some experiments, the analyzed epidermal cell sheets were all generated from the same donor, and a Mann and Whitney test was thus used. To identify differentially expressed genes or proteins in RNA-seq and proteomics experiments, respectively, paired Student’s *t*-tests were performed, and adjusted p-values were then calculated using the Benjamini–Hochberg procedure. All statistical analyses were performed using the software GraphPad Prism 4 (GraphPad Software Inc., La Jolla, CA, USA). Statistically significant differences in figures are indicated by asterisks as follows: * *p* < 0.05, ** *p* < 0.001, *** *p* < 0.0001.

### 4.11. Data Availability

The mass spectrometry proteomics data have been deposited to the ProteomeXchange Consortium via the PRIDE partner repository (https://www.ebi.ac.uk/pride/archive/) with the dataset identifier PXD01824. The RNA-seq data have been deposited on the GEO repository with the identifier GSE148985. 

## Figures and Tables

**Table 1:** Enrichment analysis of genes targeted by hydrogen-sulfide-containing compounds. The whole list of genes targeted by hydrogen-sulfide-containing compounds according to the CTD database [13] was submitted to an enrichment analysis. Enrichment in genes annotated with GO (gene ontology) terms of the “biological process” category was assessed using the enrichment analysis platform Enrichr [14]. To filter out GO terms corresponding to poorly specific biological functions, only GO terms annotating less than 100 genes were taken into account. Adjusted p-values provided by the Enrichr webtool were calculated with a modified Fisher exact test [14].

GO Term	Adjusted *p*-Value
response to reactive oxygen species	5.79 × 10^−19^
extrinsic apoptotic signaling pathway	9.14 × 10^−18^
activation of cysteine-type endopeptidase activity involved in apoptotic process	3.08 × 10^−17^
cellular response to reactive oxygen species	6.10 × 10^−17^
glutathione metabolic process	7.22 × 10^−16^
negative regulation of extrinsic apoptotic signaling pathway	2.05 × 10^−15^
cellular response to mechanical stimulus	1.00 × 10^−14^
I-kappaB kinase/NF-kappaB signaling	2.73 × 10^−13^
interleukin-1-mediated signaling pathway	2.73 × 10^−13^
toll-like receptor signaling pathway	3.46 × 10^−13^

**Table 2:** List of NaHS-modulated keratinocyte genes previously shown to be targeted by sulfide-containing compounds. The list of genes identified as being modulated by NaHS in cultured epidermal cell sheets was crossed with the list of genes previously shown to be targeted by hydrogen sulfide-containing compounds according to the CTD database [13].

Gene Symbol	Gene Name
Up-regulated genes	
*CASP1*	*Caspase-1*
*CAST*	*Calpastatin*
*CUL3*	*Cullin 3*
*CXCL2*	*C-X-C motif chemokine ligand 2*
*GDF15*	*Growth differentiation factor 15*
*NQO1*	*NAD(P)H quinone dehydrogenase 1*
*SLC3A2*	*Solute carrier family 3 member 2*
*UGT1A6*	*UDP glucuronosyltransferase family 1 member A6*
Down-regulated genes	
*CAMKK2*	*Calcium/calmodulin dependent protein kinase kinase 2*
*HOMER3*	*Homer scaffold protein 3*
*PTK2*	*Protein tyrosine kinase 2*

## Supplementary Materials

Supplementary materials can be found at https://www.mdpi.com/1422-0067/21/13/4648/s1. Figure S1. NaHS induces the intracytoplasmic accumulation of H2S in keratinocyte. Figure S2. Representative flow cytometry data obtained on dissociated cells derived from cultured human epithelial cell sheets (Kera) vs irradiated fibroblasts (Fibros) used as a feeder layer for epidermal cell sheets. Figure S3. Impact of NaHS on the cell viability of cultured human keratinocyte. Figure S4. NaHS stimulates the synthesis of SOD2 by human epidermal cell sheets. Figure S5. NaHS stimulates the synthesis of IL-8 by human epidermal cell sheets. Figure S6. NaHS treatment modifies the cytokine/chemokine profile of human epidermal cell sheets. Table S1. Mean values obtained from the analysis of dissociated epidermal cell sheets (*n* = 44) for CD49f, CD90 and HMB45. Table S2. List of proteins detected by liquid chromatography/mass spectrometry as being modulated by NaHS in human epidermal cell sheets. Table S3. List of genes targeted by hydrogen sulfide-containing compounds according to the CTD database (only protein-coding genes were retained). Table S4. List of genes displaying significant differential expression in NaHS-treated vs control epidermal cell sheets.

## Abbreviations

NAHS	Sodium hydrosulfide
H_2_S	Hydrogen sulfide
IL	Interleukin
