# Supplementary material for: Molecular Mapping of Hydrogen Sulfide Targets in Normal Human Keratinocytes
Source: Int J Mol Sci. 2020 Jun 30;21(13):4648. doi: 10.3390/ijms21134648 (PMC7369889; doi:10.3390/ijms21134648)

Supplementary material and methods

Generation of cultured human epidermal sheets

Normal human epidermal keratinocytes were isolated from human breast skin. Keratinocytes were grown on a feeder layer of irradiated human fibroblasts pre-seeded at 4000 cells /cm² in keratinocyte culture medium (KCM) containing a mix of 3:1 DMEM and HAM's F12 (Invitrogen, Carlsbad, USA), supplemented with 10% FCS, 10ng/ml epidermal growth factor (EGF; R&D systems, Minneapolis, MN, USA), 0.12 IU/ml insulin (Lilly, Saint-Cloud, France), 0.4 mg/ml hydrocortisone (UpJohn, St Quentin en Yvelelines, France) , 5 mg/ml triiodo-L-thyronine (Sigma, St Quentin Fallavier, France), 24.3 mg/ml adenine (Sigma), isoproterenol (Isuprel, Hospira France, Meudon, France) and antibiotics (20 mg/ml gentamicin (Phanpharma, Fougères, France), 100 IU/ml penicillin (Phanpharma), and 1 mg/ml amphotericin B (Phanpharma)). The medium was changed every two days. NHEK were then cultured over a period of 13 days according to the protocol currently used at the Bank of Tissues and Cells for the generation of clinical grade epidermal sheets used for the treatment of severe extended burns (Ref). When needed, cells were harvested with trypsin-EDTA 0.05% (Thermo Fisher Scientific, Waltham, MA, USA) and collected for analysis.

Clonogenic assay

Keratinocytes were seeded on a feeder layer of irradiated fibroblasts, at a clonal density of 10-20 cells/cm² and cultivated for 10 to 14 days. Three flasks per tested condition were fixed and colored in a single 30 mns step using rhodamine B (Sigma) diluted at 0.01 g/ml in 4% paraformaldehyde. In each tested condition, cells from 3 other flasks were numerated after detachment by trypsin treatment.

RNA–seq analyses

Epidermal cell sheets were generated by culturing for 12 days human keratinocytes as described above, in 6-well plates. Epidermal cell sheets were then treated or not for 24h with 0.25 mM NaHS then lysed using a RLT lysis buffer (Qiagen, Hilden, Germany) and conserved at -80°C until use. The whole RNA-seq procedure from RNA extraction to alignments of reads, data normalization, principal component analysis and identification of differentially-expressed mRNA species was performed by the Lyon-1 university genomics platform ProfileXpert and using the HiSeq 2500 platform (Illumina, 50 pb single read). To identify differentially-expressed genes, a paired student's *t*-test was performed and adjusted p-values were then calculated using the Benjamini–Hochberg procedure.

Proteomics Analysis

Epidermal cell sheets were generated by culturing for 12 days human keratinocytes as described above, in 175 cm² culture flasks. Epidermal cell sheets were then treated or not with 0.25 mM NaHS for 24h, washed two times with PBS and mechanically detached using a cell scraper. The retrieved cell suspension was centrifuged and cell pellets were conserved at -80°C until further analysis. The whole proteomics analysis from protein extraction to analysis by mass spectrometry and bioinformatics identification and quantification of proteins was performed by the University of Lille and INSERM U1192 laboratory "PRISM" (Proteomics Inflammatory response Mass Spectrometry) and using the LC-MS (liquid chromatography – mass spectrometry) apparatus Q-Exactive Orbitrap from Thermo Fisher Scientific. To identify differentially-expressed proteins, a paired student's *t*-test was performed, and adjusted p-values were then calculated using the Benjamini–Hochberg procedure.

Enzyme-linked immunosorbent assay (ELISA) protocol

Epidermal cell sheets were generated by culturing for 12 days human keratinocytes as described above, in 6-well plates. Epidermal cell sheets were then treated or not for 24h with different concentrations of NaHS. Cell supernatants were harvested and the following human cytokines were measured using ELISA kits according to the manufacturer's instructions: IL-8 (KAC1301, Biosource, Nivelles, Belgium), VEGF (DVE00, R&D systems, Minneapolis, USA), CXCL2 (ab184862, Abcam, Cambridge, MA, USA), GDF15 (EHGDF15, Thermo Fisher Scientific, Frederick, MD, USA), IL-18 (ab215539, Abcam) and IL-1β (KHC0011, Invitrogen). Alternatively, ELISA was performed on cell extracts for the measure of SOD2 (ab178012, Abcam). In any case, samples were run in duplicate.

Western blot analysis

Epidermal cell sheets were generated by culturing for 12 days human keratinocytes as described above, in 24-well plates. Epidermal cell sheets were then treated or not for 24h with different concentrations of NaHS, washed once with PBS 1X and lysed with Laemmli lysis buffer. Protein concentration was determined using the Qubit Protein

Assay Kit. Equal amounts of total protein (40µg) from treated or untreated cells were analyzed by western blot analysis. Briefly, proteins were separated in 12-15% Tris-Glycine SDS-PAGE gels and transferred to a PVDF membrane. The following primary antibodies were then applied overnight at 4°C: human proIL-1β (1:500, MAB201, Clone # 8516, R&D systems) or β-actin (1:2000, sc-47778, Clone C4, Santa Cruz Biotechnology) as a loading control. Membranes were then washed once in PBS 1X and incubated for 45 mns with horseradish peroxidase-conjugated anti-mouse IgGs (Jackson immunoresearch, Milan Analytica, La Roche, Switzerland). Finally, blots were developed using the chemiluminescent detection reagent ECL (Covalight) and exposed to X-ray films

Cell viability

Cultured human epidermal sheets treated or not with different NaHS concentrations (0.0025 – 0.025 – 0.25 – 2 – 4 mM) for 24h were trypsinized with trypsin-EDTA 0.05% (Thermo Fisher Scientific, Waltham, MA, USA) and stained with trypan blue in order to count living cells. The % of viability corresponds to the calculated ratio of unstained cells vs total cells. In another set of experiments, a MTT (3-(4,5-dimethylthiazol-2-yl)-2,5-diphenyltetrazolium bromide) assay was performed. Briefly, keratinocytes were seeded at a concentration of 8 000 cells/cm^2 in 96-well plates, on a on a feeder layer of irradiated human fibroblasts. At confluence, cells were treated or not with different concentrations of NaHS (0.0025 – 0.025 – 0.05 – 0.125 – 0.25 – 2 – 4 mM) for 24h. Absorbance measures at 565 nm were then recorded and the % of viability was calculated for each condition as the ratio of the mean absorbance observed for the treated over the mean absorbance observed for negative controls (untreated cells). Cultured keratinocytes deriving from 3 distinct donors were tested and triplicate experiments were performed for each tested condition.

Supplementary data

Figure S1. NaHS induces the intracytoplasmic accumulation of H_2S in keratinocyte. To ensure that H_2S accumulated in the cytoplasm of cultured keratinocytes following 0.25 mM NaHS stimulation, cells were sequentially incubated with an H_2S fluorescent probe and the far-red fluorescent nuclear dye NUCLEAR-ID (Enzo life Sciences, Farmingdale, NY, USA). In thelower panels (b) are shown high magnification views of the areas delineated by white line squares in the upper panels (a). Scale bar: 0.03 mm

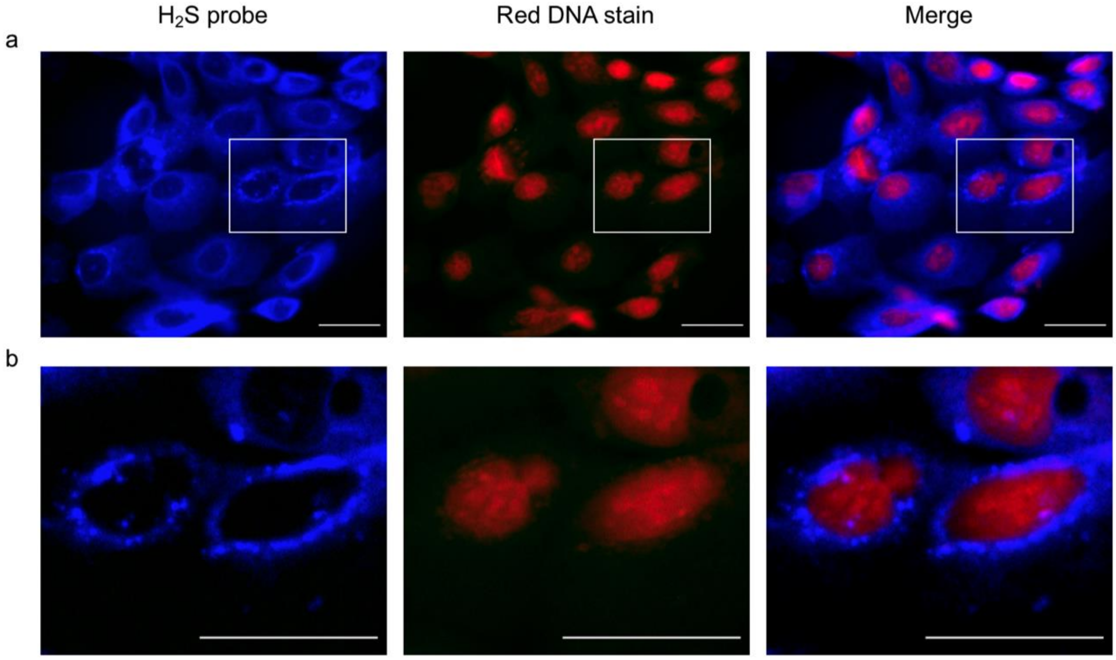

Figure S2. Representative flow cytometry data obtained on dissociated cells derived from cultured human epithelial cell sheets (Kera) vs irradiated fibroblasts (Fibros) used as a feeder layer for epidermal cell sheets. Cells derived from epidermal cell sheets exhibit a CD49^{high}/CD90^{low} phenotype whereas irradiated fibroblasts exhibit a CD90^{high}/CD49^{low} phenotype.

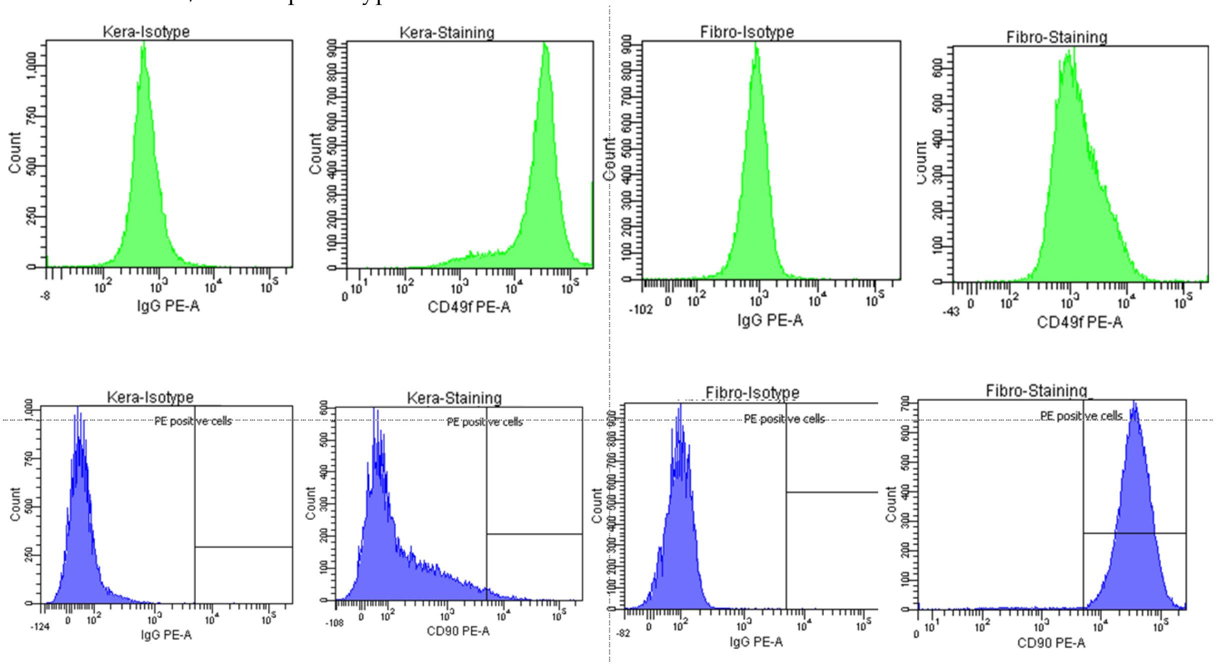

Table S1. Mean percentages of CD49f^{high}, CD90^{high} and HMB45+ cells observed from the analysis of dissociated epidermal cell sheets (n = 44)

Mean % of CD49f^{high} cells (flow cytometry)	Mean % of CD90^{high} cells (flow cytometry)	Mean % of HMB45^{+} cells (immunohistochemistry)
97.7 ± 2.2%	3.56 ± 2.8%	0%

Figure S3. Impact of NaHS on the cell viability of cultured human keratinocytes. Human epidermal cell sheets were cultured under control conditions or stimulated with NaHS at concentrations ranging from 0.0025 to 4 mM for 24h. Cells were then harvested and viability was measured after Trypan blue staining (a) (experiments performed on cells derived from 6 distinct donors) or by MTT assay (b) (experiments performed on cells derived from 3 distinct donors).

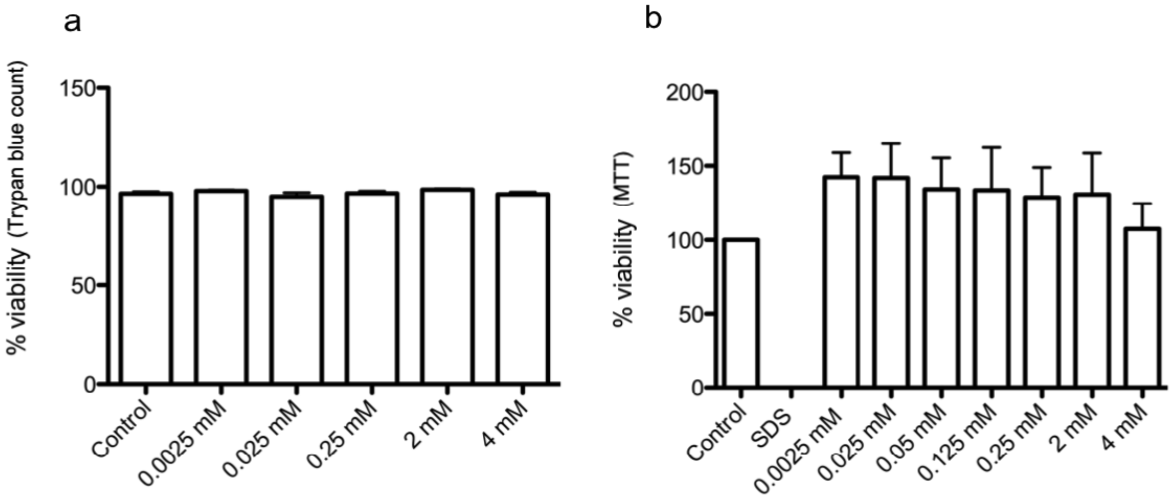

Table S2. List of proteins detected by liquid chromatography/mass spectrometry as being modulated by NaHS in human epidermal cell sheets

Protein symbol	Protein name	P-value
**Up-regulated proteins**		
FAM129B	Family with sequence similarity 129, member B	0.0133
RAB2A	Ras-related protein Rab-2A	0.0272
HNRNPU	Heterogeneous Nuclear Ribonucleoprotein U	0.0395
SND1	Staphylococcal Nuclease And Tudor Domain Containing 1	0.0399
SOD2	Superoxide Dismutase 2	0.0474
**Down-regulated proteins**		
SEC22B	SEC22 Homolog B, Vesicle Trafficking Protein	0.0160
MT-CO2	Mitochondrially Encoded Cytochrome C Oxidase I	0.0184
ALDOA	Aldolase, Fructose-Bisphosphate A	0.0331
S100A14	S100 Calcium Binding Protein A14	0.0405
LAD1	Ladinin 1	0.0487

Figure S4. NaHS stimulates the synthesis of SOD2 by human epidermal cell sheets. Human epidermal cell sheets were cultured under control conditions or were stimulated for 24h with 0.25 mM NaHS. Cells were then harvested and SOD2 was measured by ELISA on protein extracts. Experiments were performed on epidermal cell sheets obtained from 6 distinct donors. Results are expressed as percentages relative to control conditions. Statistical significance of paired comparisons was assessed with the Wilcoxon test. *: $p < 0.05$.

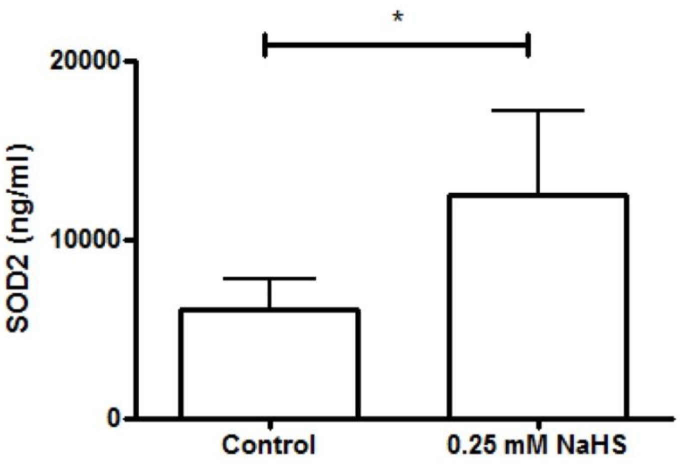

Table S3. List of genes targeted by hydrogen sulfide-containing compounds according to the CTD database (only protein-coding genes were retained)

Gene symbol							
AADAC	BRD4	CTGF	FN1	HTR1A	MGMT	PON2	SREBF1
ABCB1	BTF3	CTH	FOS	HTR1B	MIR143	PON3	SREBF2
ABCC1	BTG1	CTNNB1	FOXO3	HTR3A	MIR145	PPARA	SRSF1
ABCC2	CALR	CTSB	FOXP3	HTR7	MIR200B	PPARB	STK25
ABCC3	CAMKK2	CUL1	FOXQ1	HYOU1	MIR200C	PPARG	STX2
ABCC4	CAPN1	CUL3	FSHB	IFITM1	MIR21	PPARGC1A	SV2B
ABCC6	CAPN2	CXCL2	FTH1	IFNA1	MIR221	PPP1R12A	TAGLN2
ABCF1	CAPZA1	CXCL8	FTL	IFNGR1	MMP2	PRAM1	TERT
ABL1	CASP1	CXCR4	FTMT	IFNGR2	MMP7	PRCP	TGFB1
ACHE	CASP12	CYGB	FUBP1	IFT57	MMP9	PRDM2	TGFB3
ACOX1	CASP3	CYP19A1	G6PC	IGFBP4	MPV17	PRDX3	TGFBR2
ACTA2	CASP4	CYP1A1	G6PD	IKBKB	MSRA	PROC	TIMP1
ACTB	CASP7	CYP1A2	GADD45A	IL10	MTOR	PSMA1	TIMP2
ACTG1	CASP8	CYP1B1	GAPDH	IL1A	MYC	PSMC2	TLR10
ADORA2A	CASP9	CYP2A6	GCK	IL1B	MYD88	PSMD1	TLR3
ADRB1	CAST	CYP2B1	GCLC	IL1R2	MYH7	PTAFR	TLR4
AGT	CAT	CYP2B10	GDF15	IL1RAP	NAGLU	PTGS1	TLR6
AIFM2	CAV1	CYP2B15	GGT1	IL1RAPL2	NDRG1	PTGS2	TNF
AK2	CBL	CYP2B2	GJA1	IL1RL2	NEFH	PTK2	TNFAIP3
AKR1A1	CBS	CYP2B6	GLRX2	IL1RN	NEFL	PTP4A3	TNFAIP8
AKR7A3	CCL2	CYP2C19	GLUL	IL2	NEFM	PTPRC	TNFRSF10B
AKT1	CCL3	CYP2C9	GPT	IL2RA	NFE2L2	RARA	TNFRSF1A
ALDH1	CCNA2	CYP2D6	GRB2	IL37	NFKB2	RARB	TNFRSF1B
ALDH1A1	CCNB1	CYP2E1	GRIN1	IL4	NFKBIA	RB1	TNFSF10
ALDH1A7	CCND1	CYP3A11	GRIN2A	IL6	NLRC4	RBMX	TOLLIP
ALDH2	CCND3	CYP3A18	GSK3B	INA	NOS1	RBP1	TP53
ALDH8A1	CCNG2	CYP3A2	GSN	IRAK1	NOS2	RELA	TPT1
ALDOC	CCR3	CYP3A23	GSR	IRAK2	NOS3	RGN	TRAF6
ALOX5	CCS	CYP3A4	GSS	IRF1	NOTCH1	RGS2	TREM1
ALPL	CD14	CYP4A1	GSTA1	ITGA4	NOX4	ROCK1	TRIB3
AMH	CD55	CYP4A14	GSTA2	ITGA5	NPPA	ROCK2	TRPA1
ANXA1	CDC25C	CYP51	GSTA3	ITGAM	NPPB	RPL13A	TUBB
AOX1	CDH1	CYP7A1	GSTA4	ITGAV	NQO1	RPL17	TWIST1
APAF1	CDH5	DACH1	GSTA5	ITGB1	NR1I3	RPS27A	TXNIP
APOA1	CDK1	DDHD2	GSTM1	ITGB3	NUCB1	RPS6KB1	TXNRD1
AQP4	CDK2	DDT	GSTM2	ITGB4	ODC1	RPTOR	TXNRD2
AQP9	CDK4	DDX3Y	GSTM3	JUN	OGDH	RTN1	UBA7
AQR	CDKN1A	DEDD2	GSTM4	KCNA5	OGT	S100A11	UCHL1
AR	CDKN1B	DES	GSTP1	KCND3	OXR1	SELL	UCP1
ARHGDIB	CEBPA	DFFA	GSTT1	KCNH2	OXSR1	SERPINA1	UGT1A1

ARHGEF18	CEBPB	DHCR24	GSTZ1	KEAP1	P4HB	SERPINE1	UGT1A6
ATF3	CEBPE	DIABLO	GULO	KIF3A	PARP1	SERPINH1	UGT1A9
ATF4	CES1F	DNAJC27	HDAC1	KIT	PCBP1	SET	UGT2A3
ATF6	CES2A	DYNLRB1	HES1	KRT10	PCNA	SHC1	UGT2B35
ATG13	CFL1	EDN1	HEY1	KRT8	PDHB	SIRT1	UGT2B36
ATG14	CFLAR	EGFR	HEY2	KYAT1	PDIA3	SIRT3	UGT2B4
ATG3	CHEK1	EIF1AY	HIF1A	LAMP1	PDIA4	SKP1A	UGT3A1
ATG7	CHMP5	EIF2AK3	HIPK1	LDHA	PDYN	SLC1A2	UGT3A2
ATOX1	CHUK	EIF4E	HIST1H1C	LPA	PENK	SLC2A3P1	ULK1
ATP5PD	CLDN1	EIF4EBP1	HIST1H2BM	MAP1LC3A	PEX5	SLC38A2	USP5
BAD	CLDN11	EIF4H	HMGB1	MAP2K4	PGK1	SLC3A1	UVRAG
BAK1	CLDN2	ENO1	HMGCR	MAP2K7	PGLYRP3	SLC3A2	VCAM1
BAX	CLDN3	EPHX1	HMOX1	MAP3K3	PGM1	SLC6A4	VDAC1
BBC3	CLDN4	EVL	HNF4A	MAP3K5	PIK3R1	SLC7A11	VDAC2
BCL2	CLDN5	F2RL1	HNRNPA2B1	MAPK1	PIM1	SLC7A9	VEGFA
BCL2L1	COG3	FA2H	HNRNPC	MAPK14	PKIA	SLCO1A1	VIM
BCR	COLEC12	FADD	HNRNPDL	MAPK3	PLAUR	SNAI1	XBP1
BDNF	COX17	FAS	HNRNPH1	MAPK8	PLIN2	SNX17	XIAP
BID	CP	FASLG	HOMER3	MB	PLIN3	SOD1	XPA
BIRC2	CPT2	FASN	HSPA5	MCL1	PML	SOD2	XRCC5
BIRC3	CREB1	FBXO16	HSPA8	MDM2	POLD1	SORD	XRCC6
BIRC5	CRYL1	FBXW4P1	HSPA9	MDM4	POMC	SORL1	ZFYVE16
BMI1	CSTB	FMO1	HSPD1	MERTK	PON1	SQSTM1	ZKSCAN5

Table S4. List of genes displaying significant differential expression in NaHS-treated vs control epidermal cell sheets

Gene symbol							
**Up-regulated genes**							
ABHD12	CANX	FAM122B	LPP	NDUFA12	PPIEL	SEPT2	USP1
ABLIM2	CASP1	FARP2	LPPR2	NDUFAF4	PPP1R2P3	SHMT1	VPS45
ADRM1	CAST	FBXL4	MARVELD2	NGDN	PPP2R5E	SLC35F3	WDR31
AFAP1L2	CCDC34	FGFR1OP	MASTL	NOL8	PRC1	SLC3A2	WFS1
AKR1B10	CCDC41	FLJ44635	MCPH1	NOP58	PRTFDC1	SLC6A14	WNK1
AKR1C1	CCDC88A	GALC	MECR	NQO1	PSEN2	SLC6A15	WNT5A
ANAPC11	COL4A3BP	GDF15	MEF2D	NSA2	PSMB2	SMAD1	ZNF3
ARMCX6	COL6A3	GORASP1	MELK	NSUN2	PTPLA	SPINT1	ZNF473
ARPC3	CTSC	GPATCH11	METTL12	NUCB2	QTRTD1	SPRR2A	ZNF527
ATP5J	CUL3	GTF3C5	METTL21D	PCID2	RAB23	SYT14	ZNF778
BCAT2	CXCL2	HHAT	MPZL1	PITPNM3	RABL2B	TAF9B	
BCCIP	DAAM1	HNRNPA1	MROH1	POLG	RIF1	TLE3	
BCL2L13	DPH3	KIN	MSLN	POMT1	RIT1	TLK1	
BLM	DSTYK	KYNU	MTIF2	PPFIBP2	SCAI	TOM1	
C18orf25	DYRK4	LACTB	NCOA7	PPHLN1	SCP2	UBE2G2	
C7orf49	ERLEC1	LNPEP	NCOR2	PPHLN1	SEC11A	UGT1A6	
**Down-regulated genes**							
ABCB7	CA9	ECSIT	GORASP1	MEF2D	PPP1R3C	RUNX1	SUCO
ABTB1	CACNG4	EFEMP2	HOMER3	MGST1	PPP6R2	SCP2	SYNCRIP
ACADS	CAMKK2	ENC1	HOTAIRM1	MPDU1	PRICKLE1	SDHAP3	TCIRG1
AKIRIN1	CBY1	ENO2	IGSF8	MXD3	PRKCZ	SLC11A2	TGIF1
ANKRD37	CCDC41	FAM122B	IRF7	MYO19	PRODH	SLC16A3	TLE3
APOBEC3B	CCDC88A	FBXL4	KLK8	NDRG4	PRRX2	SLC6A11	TMC8
ARHGAP24	CDK5RAP1	FBXW7	KRT79	NDUFA4L2	PRTFDC1	SMPD1	TMEM217
BAMBI	CEPT1	FGFR1OP	LGALS1	NR1H3	PTK2	SMYD3	TRIM65
BARD1	CIRBP	FHIT	LIMA1	NR2C1	PTPN13	SNORA48	UBE2G2
BCL2L11	CPQ	FKRP	LOXL4	NTAN1	PTPRB	SPATA20	VTCN1
BNIPL	CSRNP2	FLG	LTBP3	OIP5	RABGAP1L	SPG20	WFS1
BTD	DAZAP1	FXYD5	LYNX1	PFKP	RAD51C	SPG7	WWC1
BTG2	DAZAP1	FZD8	LYPLA1	PITPNM3	RARB	SPINT1	YPEL5
C14orf159	DCAF17	GABRP	LYSMD4	PLCG1	RASGRP3	ST20	YWHAZ
C16orf13	DCAF6	GALC	MAPK7	PMS2	RCAN3	ST3GAL4	ZBTB43
C18orf56	DCAF8	GGA1	MASTL	POLG	REPIN1	STOML1	ZNF345
C19orf52	DEGS2	GIPC1	MDH1	PPHLN1	RIMS3	STX4	ZNF542
C5orf56	DLG1	GLIS2	MED8	PPP1R13L	RTN2	STXBP2	

Figure S5. NaHS stimulates the synthesis of IL-8 by human epidermal cell sheets. Human epidermal cell sheets were cultured under control conditions or stimulated with NaHS at concentrations ranging from 0.0025 mM to 4 mM. After 24h after stimulation, supernatants were harvested and cells were trypsinized in order to count viable cells. IL-8 was measured by ELISA on culture supernatants. Experiments were performed on epidermal cell sheets obtained from at least 5 distinct donors. Results are expressed as percentages relative to control conditions and adjusted to the number of viable cells. Statistical significance of paired comparisons was assessed with the Wilcoxon test. *: $p < 0.05$.

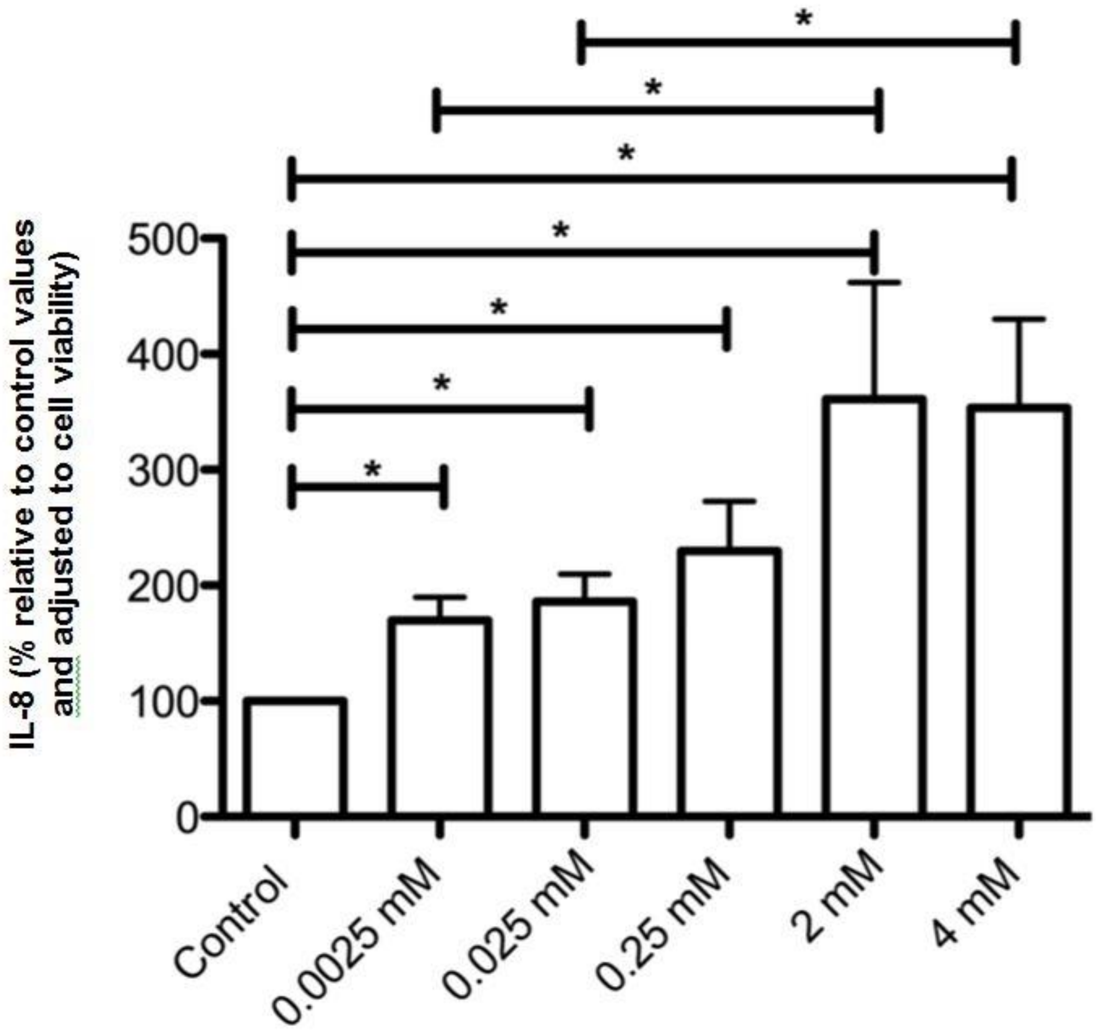

Figure S6. NaHS treatment modifies the cytokine/chemokine secretion profile of human epidermal cell sheets. Human epidermal cell sheets were cultured under control conditions or stimulated with NaHS at 0.0025, 0.025, 0.25, 2, or 4mM. In some experiments (lower right panel), cells were stimulated with NaHS (0.25 mM) and/or TNF-α (1 ng/ml). Cell supernatants were then recovered 24h after stimulation and the cytokines CXCL2, IL-1β, IL-18, VEGF and IL-8 were measured by ELISA. Data are expressed in pg/ml. Experiments were performed on epidermal cell sheets obtained from at least 5 distinct donors. Statistical significance of paired comparisons was assessed with the Wilcoxon test. *: $p < 0.05$.

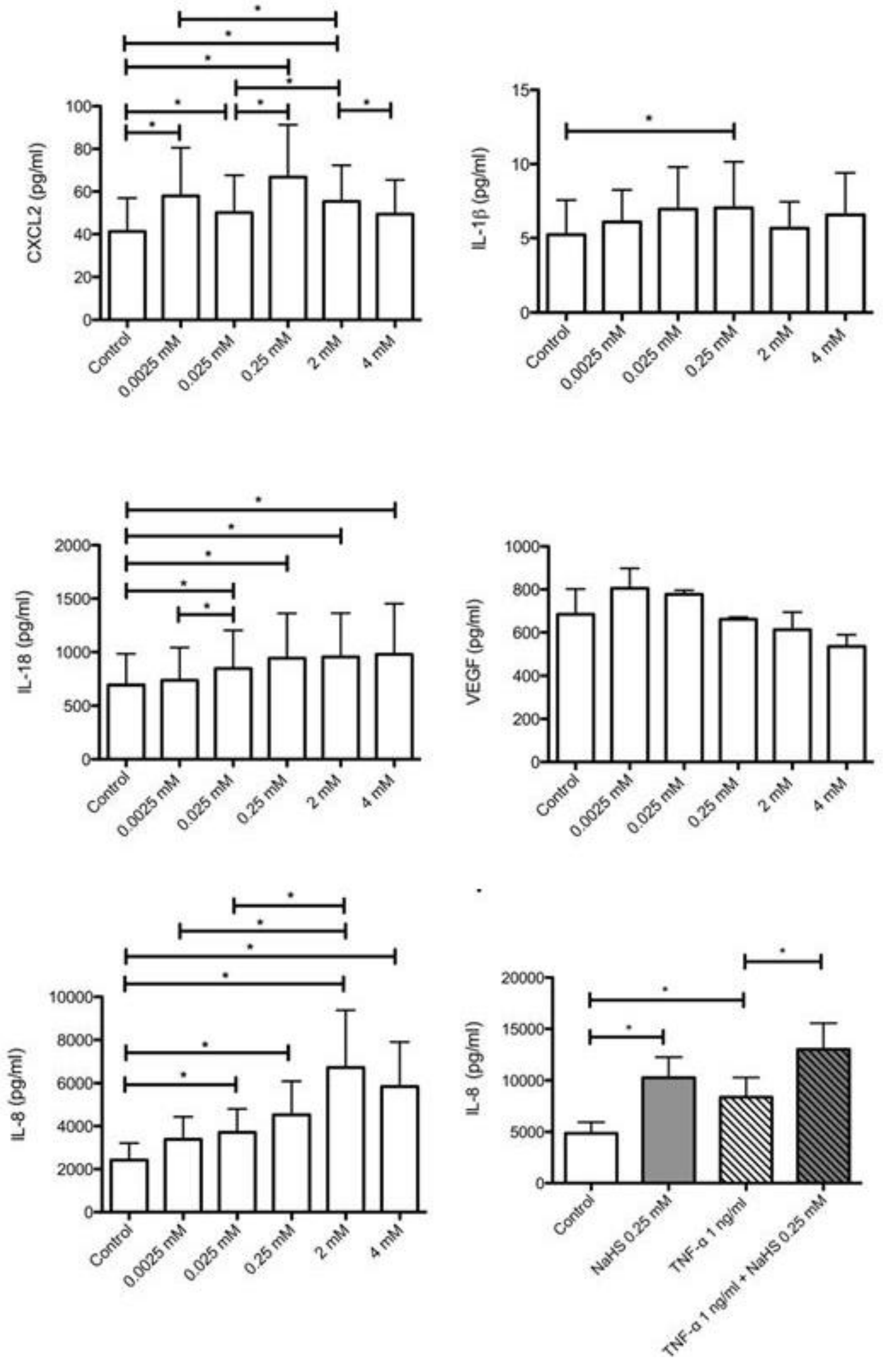

Supplement: Supplementary file 1 [file ijms-21-04648-s001.pdf]
